# Do calorie labels change energy purchased in a simulated online food delivery platform? A multi-arm randomised controlled trial

**DOI:** 10.1186/s12966-024-01638-y

**Published:** 2024-09-17

**Authors:** Madison Luick, Filippo Bianchi, Francis Bain, Lauren Bandy, Parita Doshi, Darren Hilliard, Jovita Leung, Abigail Mottershaw, Bobby Stuijfzand, Jordan Whitwell-Mak, Susan A. Jebb, Hugo Harper, Rachel Pechey

**Affiliations:** 1 Nuffield Department of Primary Care Health Sciences, University of Oxford, Oxford, UK; 2 Behavioural Insights Team, London, UK; 3 Nesta, London, UK

## Abstract

**Background:**

As rates of obesity and overweight continue to increase in the UK, calorie labels have been introduced on menus as a policy option to provide information to consumers on the energy content of foods and to enable informed choices. This study tested whether the addition of calorie labels to items in a simulated food delivery platform may reduce the energy content of items selected.

**Methods:**

UK adults (*n* = 8,780) who used food delivery platforms were asked to use the simulated platform as they would in real life to order a meal for themselves. Participants were randomly allocated to a control condition (no calorie labels) or to one of seven intervention groups: (1) large size calorie labels adjacent to the price (LP), (2) large size label adjacent to the product name (LN), (3) small label adjacent to price (SP), (4) small label adjacent to product name (SN), (5) LP with a calorie label switch-off filter (LP + Off), (6) LP with a switch-on filter (LP + On), or, (7) LP with a summary label of the total basket energy content (LP + Sum). Regression analysis assessed the impact of calorie labels on energy content of foods selected compared to the control condition.

**Results:**

The mean energy selected in the control condition was 1408 kcal (95%CI: 93, 2719). There was a statistically significant reduction in mean energy selected in five of the seven intervention trial arms (LN labels (-60 kcal, 95%CI: -111, -6), SN (-73, 95%CI: -125, -19), LP + Off (-110, 95%CI: -161, -57), LP + On (-109, 95%CI: -159, -57), LP + Sum (-85 kcal, 95%CI: -137, -30). There was no evidence the other two conditions (LP (-33, 95%CI: -88, 24) and SP (-52, 95%CI: -105, 2)) differed from control. There was no evidence of an effect of any intervention when the analysis was restricted to participants who were overweight or obese.

**Conclusion:**

Adding calorie labels to food items in a simulated online food delivery platform reduced the energy content of foods selected in five out of seven labelling scenarios. This study provides useful information to inform the implementation of these labels in a food delivery platform context.

**Supplementary Information:**

The online version contains supplementary material available at 10.1186/s12966-024-01638-y.

## Background

Rates of obesity and overweight continue to increase [1], contributing to an increase in diet-related morbidity. Reducing the overconsumption of food is a fundamental tenet of policies to prevent obesity [2].

In recent years attention has moved beyond advice on a healthy diet to consider interventions in the environments in which people select food [3] with policy interventions focused on grocery store environments. While food purchased in grocery stores accounts for the greatest proportion of total energy consumed, food purchased in the out-of-home (OOH) sector tends to have a higher energy content [4, 5] and consumption of food in the OOH sector has been associated with higher energy intake [6, 7]. A systematic review considering the use of online food retail platforms during COVID-19, including food delivery platforms, identified that the use of such platforms was connected with weight gain, increased consumption of less healthy food, and emotional eating [8]. It has also been noted that the use of food delivery platforms increases geographic access to various OOH sector outlets [9], many of which offer foods that are considered to have poor nutritional quality [9, 10]. Observational evidence points to an interaction between socioeconomic position (SEP) and the impact of takeaway consumption on energy intake, where children from lower SEP were observed to consume food from OOH outlets at home more often and to have a larger increase in energy intake than children in other groups when exposed to OOH food outlets [7, 11].

One policy tool that has been proposed to help reduce energy intake is calorie labelling. Calorie labels on menus have been introduced in various regions, including Australia in 2012 and the United States in 2018 [12–16]. Both small and null effects on energy purchases have been reported in these settings [13–17]. In April 2022, takeaway outlets in England and Wales with more than 250 employees including online and food delivery platforms were required to include calorie information on all menus [12]. One study assessing the impact of calorie labelling and proportional pricing in a virtual food delivery platform found that while calorie labelling did not impact portion sizes selected, there was an observed reduction in calories ordered from the virtual coffee shop and fast food outlet [18]. However, this experimental platform had only three possible food outlets and further research is needed to understand the impact of adding calorie labels to menus in food delivery platforms on the energy content of items selected. With the increasing use of these platforms, further evidence is needed of how calorie labels may impact ordering behaviours [19].

Previous research has shown that the effectiveness of calorie labelling depends on the prominence of this information including both font and formatting of labels [20]. In studies where less than 70% of participants noticed labels were present, there was no statistically significant change in outcomes [21]. Compliance with calorie labelling laws has also been observed to be imperfect [22, 23], and how calorie information is presented and labelling compliance enforced may be important to its effectiveness.

There may also be differential effects related to individual characteristics. For example, reported use of calorie labels is higher among people with higher education, higher income, aged > 30y, those who more frequently ate fast food and people living with overweight or obesity [24]. Alongside, there have been concerns that calorie labelling may be harmful for people with eating disorders. A hypothetical study found a significant reduction in calories selected among women with anorexia or bulimia nervosa, while those with binge eating disorder, increased calories selected [25]. However, in a university cafeteria setting where calorie labels were added, there was no evidence of adverse effects or differential behaviour changes depending on whether or not participants had an eating disorder [26].

This study aimed to assess, as a proof-of-concept, the impact of calorie labels on the energy content of food selected in a simulated food delivery platform. We aimed to test a range of presentation options to inform future policy development and industry guidance. Additionally, given the opportunities of a virtual environment and large sample size we aimed to test whether there were differences in effectiveness for population subgroups.

## Methods

### Design and recruitment

To be eligible, participants needed to be adults in the UK and users of a food delivery platform (i.e. identified that they had used a food delivery platform at least once in the past). Participants were recruited in August 2022 using Predictiv, an online policy testing platform. Quotas for age, gender, income, and location were applied to obtain a sample broadly representative of the UK population. Data from the UK Office for National Statistics was used to design quotas (See Supplementary Table 1) [27–29]. This was an eight-arm randomised controlled trial where participants were blinded to both the purpose of the study and the content of other trial arms. At the start of the study, participants were provided with an information sheet which stated ‘The aim of this study is to explore factors that influence decisions about food purchasing on online food delivery platforms’ but did not provide more information on the study details. Participants were screened for eligibility and then randomly allocated to one of eight trial arms. The random allocation was completed with a computer algorithm in the Predictiv platform after recruitment via the panel aggregator. The algorithm randomises participants at the individual level when they begin the experiment. This individual randomisation assigns each participant a random number representing one of the trial conditions. Depending on the number assigned, they see a specific version of the simulated food delivery platform in the experiment that corresponds to the intervention. This random number was then used to identify which trial arm participants had been allocated to. Randomisation was not stratified, but researchers conducted balance checks in analysis (see section *Statistics and data analysis*).

### Intervention

**Fig. 1:**
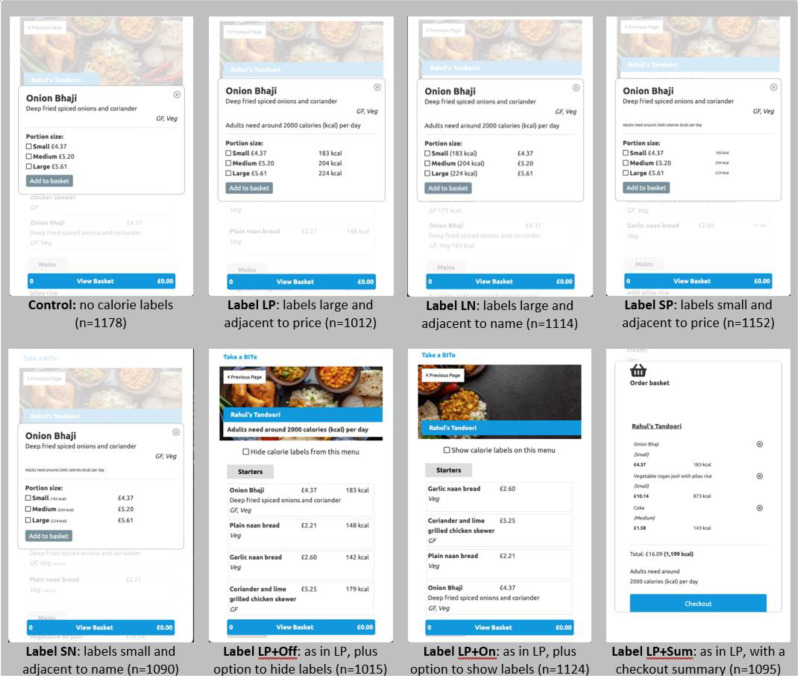
Screenshots of the study platform, showing the seven intervention trial arms with labelling conditions and the control

The study used a simulated food delivery platform, called *Take a BITe*, designed to look and function in a similar manner to real-world food delivery platforms. The platform includes 1710 individual food options, comprising 570 unique food and drink items, each offered in three different portion sizes. While the food options and restaurants (*n* = 21) are modelled on real-world food delivery platform options, these were invented for *Take a BITe* (e.g. there is no McDonalds but there are restaurants with the same type of products). The average main on *Take a BITe* contains 840 kcal and costs £8.60. For an example of how the platform appears to participants, as well as further descriptions of the platform set-up please see Bianchi et al. (Fig. 1, [30]).

**Fig. 2:**
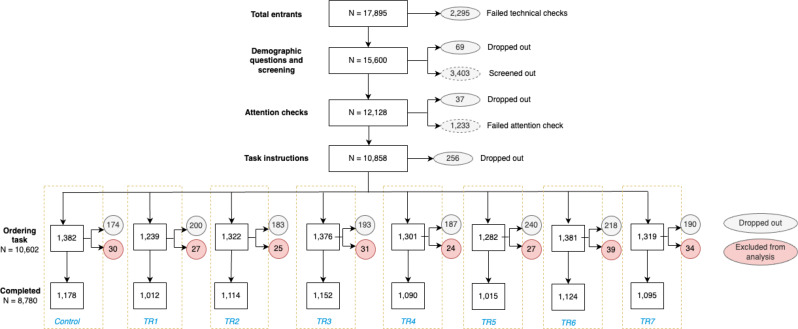
Consort flow diagram

The eight study conditions are shown in Fig. 1 and 2. The control condition had no calorie labels. In each intervention arm the energy (kcal) per serving of a small portion of food items was displayed as described below and, the statement “Adults need around 2,000 calories (kcal) per day” was displayed on the food menu page (on the right of the restaurant name) and on the portion size pop-up window (on the right of the food item’s name). Participants were unaware of the other study conditions.

1. **Large and adjacent to price (LP)** – calorie label next to price and in similar font size.
2. **Large and adjacent to product name (LN)** – the same as #1, except energy (kcal) per serving was displayed next to the description of each food item on the food menu page, and in parentheses next to the portion size in the pop-up window and checkout section. 
3. **Small and adjacent to price (SP)** – the same as #1, except the font size of the energy (kcal) per serving was 40% smaller than the price.
4. **Small and adjacent to product name (SN)** – the same as #2, except the font size of the energy (kcal) per serving was 40% smaller than the price.
5. **LP with switch off filter (LP + Off)** – the same as #1, but a filter allowing participants to hide calorie labels was available on the restaurant selection page (i.e. before participants are exposed to any calorie information - with the text “hide calorie labels from all restaurant menus”) and at the top of each food menu (with the text “hide calorie labels from this menu”).
6. **LP with switch on filter (LP + On)** – the same as #1, but a filter allowing participants to show calorie labels was available on the restaurant selection page (i.e. before participants are exposed to any calorie information - with the text “show calorie labels from all restaurant menus”) and at the top of each food menu (with the text “show calorie labels from this menu”).
7. **LP with summary calorie labels (LP + Sum)** – the same as #1, but with a basket summary at check-out, providing the total sum of energy (kcal) in the basket for all selected food items. This was displayed below the total basket price and above the check-out button in the same font size as the price along with the statement “Adults need around 2,000 calories (kcal) per day”.
8. **Control** – no calorie labels.

### Procedure

Participants were asked to complete two tasks.

In task one, participants were asked to use *Take a BITe* as they would in real life to order food for a single meal for themselves. This was considered a ‘free shopping task’, since no constraints or further instructions besides this were given to participants on what to purchase.

In task two, participants were asked to order one starter, one main, and one drink from *Take a BITe*. This was considered a ‘constrained shopping task’, since participants were instructed to limit their purchases to one of each of these items, keeping the overall energy content of items selected to a minimum, assuming they would normally purchase at least one of these items. Task two only contributed to exploratory analyses.

Following these tasks, participants were asked questions about their frequency of food delivery platform usage, height, weight, socioeconomic position, region, gender, ethnicity, education level, age, and other survey questions which contributed to exploratory analyses. Survey questions can be found in the appendix. Participants were also asked to complete the eight-item Eating Attitudes Test (EAT-8). Female participants with a score below 3, and male participants with a score below 2, were assigned to a “no eating disorder” category. Cut-offs were determined based on previous research on the EAT-8 [31]. Those with a score above this were assigned to either “binge-eating disorder” or “non-binge eating disorder” based on their answer to the question “I have gone on eating binges where I feel that I may not be able to stop”.

For both tasks, participants received no set budget. They did not spend actual money or receive the items they selected. Participants received a small financial incentive for completing the study.

### Sample size

A sample size calculation was powered to detect a 66 kcal difference based on a previous study [32] with an estimated standard deviation of 500 kcal (a Cohen’s D of 0.127), with 80% power to detect a 10% significance level. The estimated sample size was 9,000 participants, with 2,469 in the control group and 933 in each of the intervention trial arms. However, due to an error, participants were instead evenly randomised to groups. A post-hoc power calculation using achieved sample size (1,178 participants in the control group and an average of 1,086 participants in each of the intervention arms), and the same expected standard deviation and significance level (a Cohen’s D of 0.148), suggested this study was powered to detect a 74 kcal difference. The potential for a false discovery rate was controlled for using the Benjamini-Hochberg procedure, and multiple comparisons via a Bonferroni correction were taken into account in the power calculation.

### Statistics and data analysis

Exclusion criteria for participants included if their baskets contained more than 4000 kcal at the checkout, if they did not have at least 150 kcal, or if they dropped out from the experiment. These values and exclusion criteria were pre-specified. In the case where there was a duplicate identifier, only the first of these was kept. There were also attention checks to verify participants were engaging with the study, and if they did not pass, they were excluded. The attention check used was the same as in Bianchi et al. [30], and R version 4.3.2 was used to complete analyses.

#### Primary analysis

The primary outcome was the total energy (kcal) in participants’ baskets.

#### Exploratory analysis

We conducted a number of exploratory analyses, including the task two analysis, subgroup analyses for the primary outcome (i.e. by sex, SEP, BMI, food platform usage, and reported eating disorder), as well as other analyses, including on the total cost of items selected, number of items selected, calorie awareness, and support for calorie labels. All exploratory analyses are presented as the difference between the control arm and all seven treatment arms combined. Other exploratory analyses and analyses that separately investigated each trial arm, as listed in the pre-registered protocol, can be found in Supplementary Tables 2–8.

Since randomisation was not stratified, in analysis we conducted a range of balance checks which showed randomisation was balanced across all covariates (using a chi-squared test for categorical covariates). Gamma regression was used for primary analysis, price analysis, and sub-group analyses, given a right-skew was expected in the outcome variable following previous studies (and confirmed via visual assessment of the data). Linear regression was used to assess if people selected restaurants with lower calorie options, lower calorie meals, fewer foods, or smaller portion sizes. Logistic regression assessed if participants removed items from their basket or not. Linear regression was used to assess participants’ enjoyment, support, calorie awareness, and the second task. A sensitivity analysis was conducted for the primary analysis, applying a log linear model estimated with ordinary least squares (OLS).

All models in the primary analysis and exploratory analysis applied HC3 standard errors correction. All models applied Benjamini-Hochberg corrections for multiple comparisons, except for the sub-group analyses by eating disorder, since these did not involve multiple comparisons with the control as all treatment groups were pooled into one comparison against control.

### Ethics

Ethical approval was granted by The Central University Research Ethics Committee (CUREC) of the University of Oxford (R65010/RE011).

## Results

This study was completed in July and August 2022. 9,017 participants were recruited to the study and completed the task. 237 participants were excluded from the analysis because they selected food with a combined energy content below 150 kcals or above 4000 kcals, and the final analysed sample contained 8.780 participants (see CONSORT Flow Diagram and Table 1 for allocation and trial arm demographic breakdown).

**Table 1:** Proportion of respondents in each trial arm from demographic groups and mean energy purchased

	Control	LP	LN	SP	SN	LP + Off	LP + On	LP + Sum	Total
**Residence Area**									
Urban	31	33	32	31	32	31	32	29	31
Rural	18	17	17	18	18	19	18	18	18
Suburban	50	50	52	50	50	50	50	53	51
**Time of Day**									
Between 5am and 11am	38	43	38	39	40	42	38	39	39
Between 11am and 4pm	27	24	28	27	26	28	27	26	27
Between 4pm and 9pm	27	24	25	26	26	23	25	26	25
Between 9pm and 5am	9	8	9	8	9	7	9	9	9
**SEP Category**									
Low	8	6	7	7	6	7	6	6	7
High	52	53	54	54	52	54	52	53	53
Medium	40	41	39	39	42	39	42	41	40
**Frequency of Ordering**									
Every day	2	3	2	3	3	2	2	2	2
A few times a week	17	17	16	17	16	16	17	17	17
Once a week	32	29	32	28	33	31	30	32	31
Once a month	27	31	30	31	27	30	30	30	29
Less than once a month	21	21	20	22	21	21	20	19	21
**Device Used**									
Desktop	14	17	17	17	18	17	17	18	17
Mobile	86	83	83	83	82	83	83	82	83
**Location**									
London	12	13	13	12	12	14	13	12	13
Midlands	18	17	20	20	18	18	18	19	18
North	27	25	25	30	27	25	24	27	26
South and East	30	31	28	26	31	32	33	30	30
Wales, Scotland & Northern Ireland	13	14	13	12	12	12	12	12	13
**Income Category**									
Less than £30,000	54	52	54	52	52	54	52	52	53
£30,000 and over	46	48	46	48	48	46	48	48	47
**Gender**									
Male	49	48	47	50	48	48	45	47	48
Female	50	52	52	49	51	51	53	51	51
Other gender	1	0	1	1	1	1	2	2	1
**Ethnicity**									
White	86	85	84	86	85	86	84	86	85
Asian	6	7	7	7	7	6	8	7	7
Black	3	3	4	4	4	3	4	4	4
Mixed	4	4	4	3	4	4	4	3	3
Other	1	1	1	1	1	1	1	1	1
**Education Group**									
Less than high school	2	1	1	2	2	2	2	2	2
High school completed	50	49	52	53	48	49	52	50	51
University degree	47	49	46	43	49	48	45	47	47
None of the above	1	1	1	1	1	1	1	1	1
**Day of the Week**									
Sun	9	7	8	7	8	7	9	8	8
Mon	20	20	19	19	21	18	20	21	20
Tue	21	20	21	18	22	21	20	18	20
Wed	23	21	22	22	21	21	21	20	21
Thu	15	15	14	18	14	16	15	17	16
Fri	8	10	9	10	9	9	9	9	9
Sat	6	7	6	6	6	8	5	6	6
**BMI Category**									
Underweight	3	3	3	4	5	3	3	4	4
Healthy	43	42	42	42	42	44	44	45	43
Obese	24	24	24	23	24	24	21	23	23
Overweight	30	31	30	31	30	30	31	29	30
**Age Category (in years)**									
18 to 24	15	15	15	13	14	16	15	16	15
25 to 34	28	25	25	27	26	26	27	29	27
35 to 44	22	22	23	22	23	21	20	22	22
45 to 54	19	18	18	20	19	20	18	19	19
55 to 64	13	15	15	13	12	13	14	10	13
65 and over	3	5	4	5	5	5	4	4	4

Trial arms involve labelling by the following sizes, positions, and conditions: large and adjacent to price (LP), large and adjacent to product name (LN), small and adjacent to price (SP), small and adjacent to product name (SN), LP with a switch off filter (LP + off), LP with a switch on filter (LP + on), and LP with a summary calorie label (LP + Sum)

### Primary analysis

**Fig. 3:**
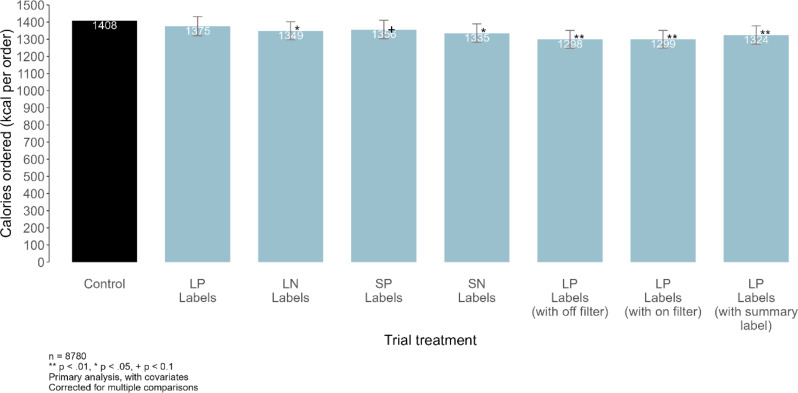
Mean energy (in kcal) from foods selected in the control group and the seven intervention groups when participants were asked to purchase food for themselves (task 1). Covariates in the models for analysis included: sex, age, income, location, education, BMI, socioeconomic position, ethnicity, residence area, time of day, frequency of ordering, and day of the week

The mean energy content of foods selected in the control was 1408 kcal (95%CI: 93, 2719) (Fig. 3). There were significant reductions in energy selected in all conditions (ranging from − 60 to -108 kcal) except where the calorie label was placed adjacent to the price where there was no evidence of an effect of the intervention (LP (-33, 95%CI: -88, 24) and SP (-52, 95%CI: -105, 2)). The same pattern and magnitude of results was observed in the sensitivity analysis using an OLS model (See Supplementary Table 2).

When analysed as all interventions compared to the control arm, the reduction in energy purchased was 75 kcal (-34 kcal, -114 kcal).

### Sub-group analyses

In subgroup analyses there was a significant intervention effect in women (-93 kcal; 95%CI: -145, -39; *n* = 4533), but not in men (-56 kcal; 95%CI: -116, 5; *n* = 4149). There was no clear trend in the effectiveness of the intervention by SEP (high SEP: -26 kcal, 95%CI: -88, 37, *n* = 3526; medium SEP: -115 kcal, 95%CI: -168,-60, *n* = 4674; low SEP: 29 kcal, 95%CI: -134, 214, *n* = 580).

The impact of labels was significant among people with BMI < 25 (-110 kcal; 95%CI: -169 kcal, -50 kcal; *n* = 4057), but not for those classed as overweight or obese (-42 kcal; 95%CI: -95 kcal, 12 kcal; *n* = 4723). There was no evidence of an effect among people classified as not having an eating disorder (-46 kcal, 95%CI: -123, 34; *n* = 1993) or with non-binge eating disorder (-42 kcal, 95%CI: -109, 28; *n* = 2989). Among people classed as having binge-eating disorder there was a significant reduction in calories selected when calorie labels were applied (-108 kcal, 95%CI: -174, -39; *n* = 3285).

There was no clear evidence labels were effective in those who used food delivery platforms regularly (-46 kcal; 95%CI: -104, 15; *n* = 4340), however, there was a significant reduction in calories selected observed in those who used food delivery platforms less than once a week (-103 kcal; 95%CI: -155, -48; *n* = 4440).

Full results by trial arm are listed in Supplementary Tables 3–7.

### Exploratory analyses

**Fig. 4:**
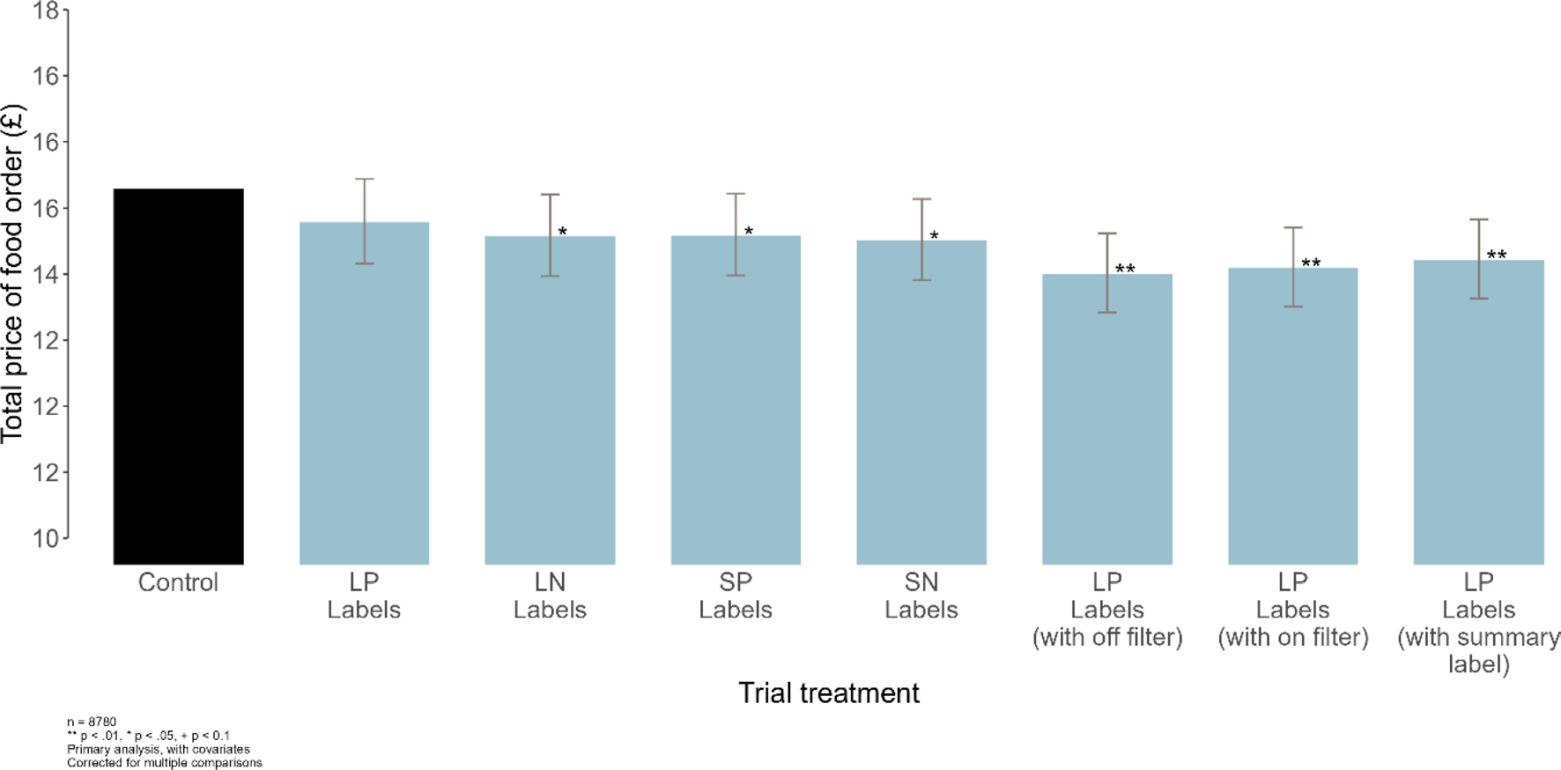
Mean money spent (£) in the control and seven intervention groups for task 1. Covariates in the models for analysis included: sex, age, income, location, education, BMI, socioeconomic position, ethnicity, residence area, time of day, frequency of ordering, and day of the week

Compared to the control, slightly fewer items were selected in conditions with labels (-0.06 items, *p* = 0.0012) and the total price of foods selected was slightly lower (-£0.89, *p* = 0.0002). Fig. 4 shows the total price of food by trial arm.

Participants did not report greater awareness of the energy content of their food when calorie labels were present (*p* = 0.110), and when calorie awareness was analysed by trial arms, only participants in the LN trial arm were shown to have increased calorie awareness compared to control (*p* = 0.016). Other exploratory outcomes, including task two, are reported in Supplementary Tables 8–9.

## Discussion

Applying labels with calorie information to menus in a simulated food delivery platform reduced the energy content of foods selected by participants. When analysed by labelling type, five out of seven study conditions were observed to have a significant impact on reducing the energy content of foods selected. The greatest reduction in energy content was seen in the interventions that allowed an on or off filter for the calorie label information. No labelling conditions resulted in an increase in energy content of foods selected. Exploratory analyses found the number of items and the total cost of items selected decreased in intervention groups. There were reductions in energy selected in all subgroup analyses, though not all were significant. Most notably there was a significant effect in women but not men, and in people with a healthy BMI but not those living with overweight or obesity, and among people identified as possibly having a binge-eating disorder. This indicates calorie labels may be effective for some population groups, but potentially less effective for populations already living with overweight or obesity.

One of the many strengths of this study is its use of a randomised controlled trial design and large sample size to systematically test different labelling designs. It provides proof-of-concept evidence that calorie labels may be effective at reducing the energy content of food selected in the novel context of a food delivery platform. This was a simulated food delivery platform, but the energy content of food selected (i.e. 1408 kcal in the control) was similar to the reported energy content of real takeaway food [33]. The simulated platform allowed for greater flexibility in testing a range of potential labelling options. However, as this was a simulated environment, participants were not spending their own money or receiving the food they selected and items were positioned at random and not promoted, which is not the case in real food delivery platforms [34]. A study based in the US has found that the implementation of calorie labels to menus did not negatively impact restaurant revenues [35]; a key potential aspect of calorie labelling policies from the perspective of restaurants. When participants are not spending their own money, however, it is difficult to robustly draw conclusions on these potential impacts. Research has also shown that the implementation of kilojoule labelling legislation in Australia did not result in perfect compliance in food delivery platforms [22], and more generally in the OOH sector in England, only 15% of outlets were found to be in full compliance with the new calorie labelling legislation [23]. This suggests a real food delivery platform may not display calorie labelling information as consistently as shown in this study, likely reducing effectiveness. The nature of the intervention, adding calorie labels, means that participants in these trial arms may have been aware of what was being researched, and social desirability bias may have influenced participants’ selections or the extent of impact of labels. Moreover, this study was conducted in August 2022, and calorie labels were implemented in England in April 2022, meaning some participants may have had previous exposure to calorie labels in real-world food delivery platforms. We are not able to determine whether previous exposure may impact responses, although responsiveness following longer-term exposure is of primary interest in terms of sustained public health impacts and should be a focus of future research. While this study shows calorie labels may have some effect when tested in a robust research design, field trials will be needed to understand if this effect replicates outside of a simulated context.

To avoid multiple additional comparisons, we did not directly compare interventions with each other, so impressions of the comparative effectiveness are tentative. Interventions with a filter or summary label had the greatest impact compared to control, followed by those that listed the label adjacent to the product. Those that had the label adjacent to the price, and no filter or summary label, had no observed effect. Our hypothesis is that this may have been due to the salience of the label and maybe even more so, the point in the decision-making process when the labels were noticed; being more effective when the label was adjacent to the product rather than the price (with energy content likely to therefore be seen before price, reading left-to-right, in this context). As calorie information starts to be added to food delivery platforms, in the absence of standardised policies or consistency of applying labels [36], further research is needed to identify the most effective presentation of calorie information.

As previously reported some subgroups of the population may be more sensitive to calorie labelling interventions, notably women more than men [24], but broadly we showed a population wide reduction in energy selected in response to the intervention. There was no clear trend by SEP, which if replicated, may not support the hypothesis that labelling interventions could increase inequalities [37]. However, the direction of effects, if consistent in a larger powered study, would also not be inconsistent with this hypothesis, suggesting more research should be done on the differential effects of calorie labels based on individual health literacy or the degree to which an individual is health conscious. Significant reductions in energy selected among people of a healthy weight but no significant change (a point estimate suggesting any effect was half the magnitude) in those living with overweight or obesity, was unexpected. Findings in relation to possible eating disorders were complex and require further investigation, not least since the classification was based on responses to a questionnaire, and not self-reported or clinically diagnosed eating disorders.

This study recruited a sample that was representative of the UK population, however, research has shown that the odds of using food delivery platforms may vary by sociodemographic factors, with one study finding that being younger, male, highly educated, living with children, or identifying with an ethnic minority all increased the odds of using food delivery platforms [38]. This would be an important consideration in estimating the population-level impact that may accrue from full implementation. For the five significant reductions reported here, all represented larger reductions in energy selected than those applied in modelling the impacts of introducing calorie labelling in the OOH sector, which applied a 47 kcal reduction for each out-of-home meal, and found an estimated 0.31% reduction in obesity prevalence over 20 years [39]. While this indicates promise in terms of potential (albeit small) impact, given this was a proof-of-concept study, effect sizes from this study would not be expected to translate directly to real-world scenarios and testing on actual food delivery platforms is needed.

## Conclusions

This proof-of-concept study shows that calorie labels in food delivery platforms can support people to make lower calorie food selections; albeit within a simulated environment, and using a one-off task. Nonetheless it provides useful information to inform the implementation of these labels in a food delivery platform context.

## Electronic supplementary material

Below is the link to the electronic supplementary material.

Supplementary Material 1

Supplementary Material 2

Supplementary Material 3

## Data Availability

Data will be made available upon request.

## Abbreviations

BMI: Body mass index
EAT 8: 8 item Eating Attitudes Test
LN: label condition with large labels presented adjacent to product name
LP: Label condition with large labels presented adjacent to price
LP + Off: Label condition with large labels presented adjacent to price, with a switch off filter
LP + On: Label condition with large labels presented adjacent to price, with a switch on filter
LP + Sum: Label condition with large labels presented adjacent to price, with a summary calorie label
OLS: Ordinary least squares
OOH: Out–of–home
SEP: Socioeconomic position
SN: Label condition with small labels presented adjacent to product name
SP: Label condition with small labels presented adjacent to price

## References

[1] NHS Digital. Statistics on Obesity, Physical Activity and Diet, England. 2020.

[2] Public Health England. Calorie reduction: the scope and ambition for action. 2019.

[3] Marteau TM, Hollands GJ, Fletcher PC. Changing human behavior to prevent disease: the importance of targeting automatic processes. Science. 2012;337(6101):1492–5. 22997327 10.1126/science.1226918

[4] Muc M, Jones A, Roberts C, Sheen F, Haynes A, Robinson E. A bit or a lot on the side? Observational study of the energy content of starters, sides and desserts in major UK restaurant chains. BMJ Open. 2019;9(10):e029679. 31594875 10.1136/bmjopen-2019-029679 PMC6797243

[5] Robinson E, Jones A, Whitelock V, Mead BR, Haynes A. (Over)eating out at major UK restaurant chains: observational study of energy content of main meals. BMJ. 2018;363:k4982. 30541906 10.1136/bmj.k4982 PMC6290483

[6] Burgoine T, Forouhi NG, Griffin SJ, Wareham NJ, Monsivais P. Associations between exposure to takeaway food outlets, takeaway food consumption, and body weight in Cambridgeshire, UK: population based, cross sectional study. BMJ. 2014;348:g1464. 24625460 10.1136/bmj.g1464 PMC3953373

[7] Goffe L, Rushton S, White M, Adamson A, Adams J. Relationship between mean daily energy intake and frequency of consumption of out-of-home meals in the UK National Diet and Nutrition Survey. Int J Behav Nutr Phys Act. 2017;14(1):131. 28938893 10.1186/s12966-017-0589-5 PMC5610411

[8] Jia SS, Raeside R, Sainsbury E, Wardak S, Phongsavan P, Redfern J, et al. Use of online food retail platforms throughout the COVID-19 pandemic and associated diet-related chronic disease risk factors: a systematic review of emerging evidence. Obes Rev. 2024;25(6):e13720. 38346847 10.1111/obr.13720

[9] Brar K, Minaker LM. Geographic reach and nutritional quality of foods available from mobile online food delivery service applications: novel opportunities for retail food environment surveillance. BMC Public Health. 2021;21(1):458. 33676458 10.1186/s12889-021-10489-2 PMC7937239

[10] Horta PM, Souza JPM, Rocha LL, Mendes LL. Digital food environment of a Brazilian metropolis: food availability and marketing strategies used by delivery apps. Public Health Nutr. 2021;24(3):544–8. 32900419 10.1017/S1368980020003171 PMC10195591

[11] Adams J, Goffe L, Brown T, Lake AA, Summerbell C, White M, et al. Frequency and socio-demographic correlates of eating meals out and take-away meals at home: cross-sectional analysis of the UK national diet and nutrition survey, waves 1–4 (2008–12). Int J Behav Nutr Phys Activity. 2015;12(1):51. 10.1186/s12966-015-0210-8 PMC4404110 25889159

[12] New calorie labelling. Rules come into force to improve nation’s health [press release]. GOV.UK, 6 April 2022 2022.

[13] NSW Food Authority. Evaluation of kilojoule menu labelling. NSW Food Authority; 2013.

[14] Petimar J, Zhang F, Cleveland LP, Simon D, Gortmaker SL, Polacsek M, et al. Estimating the effect of calorie menu labeling on calories purchased in a large restaurant franchise in the southern United States: quasi-experimental study. BMJ. 2019;367:l5837. 31666218 10.1136/bmj.l5837 PMC6818731

[15] Petimar J, Zhang F, Rimm EB, Simon D, Cleveland LP, Gortmaker SL, et al. Changes in the calorie and nutrient content of purchased fast food meals after calorie menu labeling: a natural experiment. PLoS Med. 2021;18(7):e1003714. 34252088 10.1371/journal.pmed.1003714 PMC8312920

[16] Zlatevska N, Neumann N, Dubelaar C. Mandatory calorie Disclosure: a comprehensive analysis of its effect on consumers and retailers. J Retail. 2018;94(1):89–101.

[17] Cantor J, Torres A, Abrams C, Elbel B. Five years later: awareness of New York City’s calorie labels declined, with no changes in calories purchased. Health Aff (Millwood). 2015;34(11):1893–900. 26526247 10.1377/hlthaff.2015.0623

[18] Finlay A, Boyland E, Jones A, Witkam R, Robinson E. The impact of calorie labelling and proportional pricing on out of home food orders: a randomised controlled trial study using a virtual food and drink delivery app. Int J Behav Nutr Phys Activity. 2023;20(1):112. 10.1186/s12966-023-01513-2 PMC10508026 37726788

[19] Statista. Online Food Delivery - United Kingdom. 2022 [ https://www.statista.com/outlook/dmo/online-food-delivery/united-kingdom

[20] Acton RB, Vanderlee L, White C, Hammond D. The efficacy of calorie labelling formats on pre-packaged foods: an experimental study among adolescents and young adults in Canada. Can J Public Health. 2016;107(3):e296–302. 27763846 10.17269/CJPH.107.5513 PMC6972474

[21] Littlewood JA, Lourenco S, Iversen CL, Hansen GL. Menu labelling is effective in reducing energy ordered and consumed: a systematic review and meta-analysis of recent studies. Public Health Nutr. 2016;19(12):2106–21. 26714776 10.1017/S1368980015003468 PMC10270829

[22] Cassano S, Jia A, Gibson AA, Partridge SR, Chan V, Farrell P, et al. Benchmarking online food delivery applications against menu labelling laws: a cross-sectional observational analysis. Public Health Nutr. 2024;27(1):e101. 38557393 10.1017/S1368980024000673 PMC11036439

[23] Polden M, Jones A, Essman M, Adams J, Bishop T, Burgoine T, et al. Point-of-choice kilocalorie labelling practices in large, out-of-home food businesses: a preobservational versus post observational study of labelling practices following implementation of the calorie labelling (out of Home Sector) (England) regulations 2021. BMJ Open. 2024;14(4):e080405. 38604637 10.1136/bmjopen-2023-080405 PMC11015320

[24] Breck A, Cantor J, Martinez O, Elbel B. Who reports noticing and using calorie information posted on fast food restaurant menus? Appetite. 2014;81:30–6. 24882449 10.1016/j.appet.2014.05.027 PMC4127350

[25] Haynos AF, Roberto CA. The effects of restaurant menu calorie labeling on hypothetical meal choices of females with disordered eating. Int J Eat Disord. 2017;50(3):275–83. 28130796 10.1002/eat.22675 PMC5378635

[26] Lillico HG, Hanning R, Findlay S, Hammond D. The effects of calorie labels on those at high-risk of eating pathologies: a pre-post intervention study in a University cafeteria. Public Health. 2015;129(6):732–9. 25931435 10.1016/j.puhe.2015.03.005

[27] Office for National Statistics. Average household income, UK: financial year ending 2019; 2020.

[28] Office for National Statistics. Estimates of the population for the UK, England, Wales, Scotland and Northern Ireland; 2020.

[29] Office for National Statistics. Ethnicity facts and figures: Age groups. 2020. Available from: https://www.ethnicity-factsfigures.service.gov.uk/uk-population-by-ethnicity/demographics/age-groups/latest

[30] Bianchi F, Luick M, Bandy L, Bone J, Kelly S, Farrington J, et al. The impact of altering restaurant and menu option position on food selected from an experimental food delivery platform: a randomised controlled trial. Int J Behav Nutr Phys Act. 2023;20(1):60. 37208720 10.1186/s12966-023-01456-8 PMC10197857

[31] Richter F, Strauss B, Braehler E, Altmann U, Berger U. Psychometric properties of a short version of the Eating Attitudes Test (EAT-8) in a German representative sample. Eat Behav. 2016;21:198–204 10.1016/j.eatbeh.2016.03.006 26978119

[32] Bone J, Arias A, Farrington J, Leung J, Bianchi F, Harper H. Reducing the calories in takeaway orders Nesta. 2022. Available from: https://www.nesta.org.uk/project-updates/reducing-calories-takeaway-orders/

[33] Jaworowska A, Blackham M, Long T, Taylor R, Ashton C, Stevenson M. Nutritional composition of takeaway food in the UK. Nutr Food Sci. 2014;44(5):414–30.

[34] Horta PM, Souza JPM, Mendes LL. Food promoted on an online food delivery platform in a Brazilian metropolis during the COVID-19 pandemic: a longitudinal analysis. Public Health Nutr. 2022;25(5):1–23. 35232512 10.1017/S1368980022000489 PMC9043632

[35] Susskind A, Willage B, Cawley J. The impact of Restaurant Menu calorie labels on Restaurant revenue and profit: evidence from a Randomized Controlled Trial. Cornell Hospitality Q. 2023;65(2):227–39.

[36] Vanderlee L, Gaucher-Holm A, Le-Brassard M, Vaillancourt C. Availability of calorie information on online food delivery service platforms among major chain restaurants in Canadian provinces with different calorie labelling policies. Can J Public Health. 2023;114(6):983–991. 10.17269/s41997-023-00788-z PMC10726687 37386269

[37] Adams J, Mytton O, White M, Monsivais P. Why are some Population interventions for Diet and obesity more Equitable and Effective Than others? The role of Individual Agency. PLoS Med. 2016;13(4):e1001990. 27046234 10.1371/journal.pmed.1001990 PMC4821622

[38] Keeble M, Adams J, Sacks G, Vanderlee L, White CM, Hammond D, et al. Use of online food delivery services to order food prepared away-from-home and associated sociodemographic characteristics: a cross-sectional, multi-country analysis. Int J Environ Res Public Health. 2020;17(14):5190. 10.3390/ijerph17145190 PMC7400536 32709148

[39] Colombet Z, Robinson E, Kypridemos C, Jones A, O’Flaherty M. Effect of calorie labelling in the out-of-home food sector on adult obesity prevalence, cardiovascular mortality, and social inequalities in England: a modelling study. Lancet Public Health. 2024;9(3):e178–85. 38429017 10.1016/S2468-2667(23)00326-2

