# Supplementary material for: Do calorie labels change energy purchased in a simulated online food delivery platform? A multi-arm randomised controlled trial
Source: Int J Behav Nutr Phys Act. 2024 Sep 17;21:103. doi: 10.1186/s12966-024-01638-y (PMC11406871; doi:10.1186/s12966-024-01638-y)
Supplement: Supplementary file 1 — Supplementary Material 1 [file 12966_2024_1638_MOESM1_ESM.docx]

Supplementary Table 1. Quotas used in recruitment of participants to the study.

|  | ***Target (%)*** | ***Sample (%)*** | ***Source*** |
| --- | --- | --- | --- |
| ***Age*** |  |  |  |
| *18-24* | *12%* | *15%* | [*ONS*](https://www.ons.gov.uk/peoplepopulationandcommunity/populationandmigration/populationestimates/datasets/analysisofpopulationestimatestool) |
| *25-34* | *18%* | *27%* |  |
| *35-44* | *16%* | *22%* |  |
| *45-54* | *18%* | *19%* |  |
| *55-64* | *16%* | *13%* |  |
| *65+* | *24%* | *4%* |  |
| ***Gender*** |  |  | *-* |
| *Female* | *50%* | *51%* |  |
| *Male* | *50%* | *48%* |  |
| *Other* | *-* | *1%* |  |
| ***Income*** |  |  | [*ONS*](https://www.ons.gov.uk/peoplepopulationandcommunity/personalandhouseholdfinances/incomeandwealth/bulletins/householddisposableincomeandinequality/financialyearending2021) *+* [*Money Saving Expert.*](https://www.moneysavingexpert.com/tax-calculator/) |
| *Above £40,000* | *50%* | *47%* |  |
| *Below $40,000* | *50%* | *53%* |  |
| ***Location*** |  |  | [*ONS*](https://www.ons.gov.uk/peoplepopulationandcommunity/populationandmigration/populationestimates/datasets/analysisofpopulationestimatestool) |
| *North* | *23%* | *26%* |  |
| *South & East* | *32%* | *30%* |  |
| *Midlands* | *16%* | *18%* |  |
| *London* | *13%* | *13%* |  |
| *Scotland, Wales & NI* | *16%* | *13%* |  |
| ***Ethnicity*** |  |  | [*Gov.uk*](https://www.ethnicity-facts-figures.service.gov.uk/uk-population-by-ethnicity/demographics/age-groups/latest) |
| *White* | *86%* | *85%* |  |
| *Ethnic minority* | *14%* | *15%* |  |

Supplementary Table 2. Model estimates and change relative to the control group mean for the primary analysis and price analysis.

| Primary Analysis: 8 trial arms | | | | | | | | | | |
| --- | --- | --- | --- | --- | --- | --- | --- | --- | --- | --- |
| Trial Group | | Estimate | | Mean | | 95% CI (from exponentiated estimate) | | | P-value | Q-value |
| Control | | NA | | 1408 | | NA | | NA | NA | NA |
| LARGE AND ADJACENT TO PRICE | | -0.024 | | 1375 | | 1320 | | 1432 | 0.255 | 0.255 |
| LARGE AND ADJACENT TO PRODUCT | | -0.043 | | 1349 | | 1297 | | 1402 | 0.029 | 0.040 |
| SMALL AND ADJACENT TO PRICE | | -0.038 | | 1356 | | 1302 | | 1411 | 0.062 | 0.073 |
| SMALL AND ADJACENT TO PRODUCT | | -0.053 | | 1335 | | 1283 | | 1389 | 0.009 | 0.015 |
| LP WITH SWITCH OFF FILTER | | -0.081 | | 1298 | | 1248 | | 1352 | <0.001 | <0.001 |
| LP WITH SWITCH ON FILTER | | -0.080 | | 1299 | | 1249 | | 1352 | <0.001 | <0.001 |
| LP WITH SUMMARY CALORIE LABEL | | -0.062 | | 1324 | | 1272 | | 1378 | 0.002 | 0.006 |
| Primary Analysis: control versus all labelling trial arms combined | | | | | | | | | | |
| Trial Group | | Estimate | | Mean | | 95% CI (from exponentiated estimate) | | | P-value | Q-value |
| Control | |  | | 1408 | | NA | | NA | NA | NA |
| ALL LABELLING TREATMENTS | | -0.054 | | 1334 | | 1295 | | 1374 | <0.001 | <0.001 |
| Price Analysis: 8 trial arms | | | | | | | | | | |
| Trial Group | Estimate | | Mean | | 95% CI (relative to control mean) | | | | P-value | Q-value |
| Control |  | | 15.29 | | NA | | NA | | NA | NA |
| LARGE AND ADJACENT TO PRICE | -0.03 | | 14.79 | | 14.16 | | 15.44 | | 0.129 | 0.129 |
| LARGE AND ADJACENT TO PRODUCT | -0.05 | | 14.57 | | 13.97 | | 15.20 | | 0.027 | 0.032 |
| SMALL AND ADJACENT TO PRICE | -0.05 | | 14.58 | | 13.98 | | 15.21 | | 0.028 | 0.032 |
| SMALL AND ADJACENT TO PRODUCT | -0.05 | | 14.51 | | 13.91 | | 15.14 | | 0.016 | 0.025 |
| LP WITH SWITCH OFF FILTER | -0.09 | | 14.00 | | 13.42 | | 14.62 | | <0.001 | <0.001 |
| LP WITH SWITCH ON FILTER | -0.08 | | 14.10 | | 13.51 | | 14.71 | | <0.001 | <0.001 |
| LP WITH SUMMARY CALORIE LABEL | -0.07 | | 14.22 | | 13.63 | | 14.83 | | 0.001 | 0.001 |
| Price Analysis: control versus all labelling trial arms combined | | | | | | | | | | |
| Trial Group | Estimate | | Mean | | 95% CI (Marginal) | | | | P-value | Q-value |
| Control |  | | 15.29 | | NA | | NA | | <0.001 | <0.001 |
| ALL LABELLING TREATMENTS | -0.060 | | 14.39 | | 13.94 | | 14.86 | | <0.001 | <0.001 |
| Sensitivity Analysis (log-linear) of Primary Analysis: 8 trial arms | | | | | | | | | | |
| Trial Group | Estimate | | Mean | | 95% CI (from estimate) | | | | P-value | Q-value |
| Control | NA | | 1408 | | NA | | NA | | NA | NA |
| LARGE AND ADJACENT TO PRICE | -28 | | 1380 | | 1324 | | 1436 | | 0.332 | 0.332 |
| LARGE AND ADJACENT TO PRODUCT | -58 | | 1350 | | 1297 | | 1403 | | 0.032 | 0.045 |
| SMALL AND ADJACENT TO PRICE | -51 | | 1357 | | 1303 | | 1411 | | 0.066 | 0.077 |
| SMALL AND ADJACENT TO PRODUCT | -70 | | 1338 | | 1284 | | 1393 | | 0.012 | 0.021 |
| LP WITH SWITCH OFF FILTER | -106 | | 1302 | | 1248 | | 1355 | | <0.001 | <0.001 |
| LP WITH SWITCH ON FILTER | -105 | | 1303 | | 1250 | | 1356 | | <0.001 | <0.001 |
| LP WITH SUMMARY CALORIE LABEL | -83 | | 1325 | | 1271 | | 1379 | | <0.001 | <0.001 |

Supplementary Table 3. Model estimates and change relative to the control group mean for SEP sub-group analyses.

| Sub-group Analysis: Low SEP, 8 trial arms | | | | | | | |
| --- | --- | --- | --- | --- | --- | --- | --- |
| Trial Group | Estimate | Mean | | 95% CI (from exponentiated estimate) | | P-value | Q-value |
| Control |  | 1387 | NA | | NA | NA | NA |
| LARGE AND ADJACENT TO PRICE | 0.111 | 1550 | 1293 | | 1858 | 0.229 | 0.917 |
| LARGE AND ADJACENT TO PRODUCT | -0.022 | 1356 | 1168 | | 1575 | 0.770 | 0.943 |
| SMALL AND ADJACENT TO PRICE | -0.006 | 1378 | 1163 | | 1633 | 0.943 | 0.943 |
| SMALL AND ADJACENT TO PRODUCT | -0.038 | 1335 | 1125 | | 1584 | 0.662 | 0.943 |
| LP WITH SWITCH OFF FILTER | 0.012 | 1404 | 1201 | | 1640 | 0.879 | 0.943 |
| LP WITH SWITCH ON FILTER | 0.066 | 1482 | 1259 | | 1744 | 0.427 | 0.943 |
| LP WITH SUMMARY CALORIE LABEL | 0.024 | 1420 | 1203 | | 1677 | 0.780 | 0.943 |
| Low SEP Sub-group Analysis: control versus all labelling trial arms combined | | | | | | | |
| Trial Group | Estimate | Mean | | 95% CI (from exponentiated estimate) | | P-value | Q-value |
| Control |  | 1387 | NA | | NA | NA | NA |
| ALL LABELLING TREATMENTS | 0.021 | 1416 | 1252 | | 1601 | 0.741 | 0.741 |
| Sub-group Analysis: Medium SEP, 8 trial arms | | | | | | | |
| Trial Group | Estimate | Mean | | 95% CI (from exponentiated estimate) | | P-value | Q-value |
| Control |  | 1427 | NA | | NA | NA | NA |
| LARGE AND ADJACENT TO PRICE | -0.074 | 1325 | 1255 | | 1399 | 0.007 | 0.010 |
| LARGE AND ADJACENT TO PRODUCT | -0.059 | 1345 | 1275 | | 1419 | 0.030 | 0.030 |
| SMALL AND ADJACENT TO PRICE | -0.071 | 1329 | 1258 | | 1404 | 0.011 | 0.013 |
| SMALL AND ADJACENT TO PRODUCT | -0.087 | 1309 | 1239 | | 1383 | 0.002 | 0.003 |
| LP WITH SWITCH OFF FILTER | -0.096 | 1296 | 1227 | | 1370 | 0.001 | 0.001 |
| LP WITH SWITCH ON FILTER | -0.103 | 1287 | 1220 | | 1358 | <0.001 | 0.001 |
| LP WITH SUMMARY CALORIE LABEL | -0.099 | 1293 | 1224 | | 1365 | <0.001 | 0.001 |
| Medium SEP Sub-group Analysis: control versus all labelling trial arms combined | | | | | | | |
| Trial Group | Estimate | Mean | | 95% CI (from exponentiated estimate) | | P-value | Q-value |
| Control |  | 1427 | NA | | NA | NA | NA |
| ALL LABELLING TREATMENTS | -0.084 | 1313 | 1260 | | 1368 | <0.001 | <0.001 |
| Sub-group Analysis: High SEP, 8 trial arms | | | | | | | |
| Trial Group | Estimate | Mean | | 95% CI (from exponentiated estimate) | | P-value | Q-value |
| Control |  | 1387 | NA | | NA | NA | NA |
| LARGE AND ADJACENT TO PRICE | 0.026 | 1422 | 1332 | | 1519 | 0.446 | 0.769 |
| LARGE AND ADJACENT TO PRODUCT | -0.016 | 1365 | 1283 | | 1451 | 0.613 | 0.769 |
| SMALL AND ADJACENT TO PRICE | 0.011 | 1402 | 1317 | | 1493 | 0.725 | 0.769 |
| SMALL AND ADJACENT TO PRODUCT | -0.009 | 1374 | 1291 | | 1462 | 0.769 | 0.769 |
| LP WITH SWITCH OFF FILTER | -0.066 | 1298 | 1219 | | 1384 | 0.042 | 0.113 |
| LP WITH SWITCH ON FILTER | -0.069 | 1294 | 1215 | | 1378 | 0.031 | 0.113 |
| LP WITH SUMMARY CALORIE LABEL | -0.014 | 1368 | 1282 | | 1458 | 0.671 | 0.769 |
| High SEP Sub-group Analysis: control versus all labelling trial arms combined | | | | | | | |
| Trial Group | Estimate | Mean | | 95% CI (from exponentiated estimate) | | P-value | Q-value |
| Control |  | 1387 | NA | | NA | NA | NA |
| ALL LABELLING TREATMENTS | -0.020 | 1360 | 1299 | | 1424 | 0.404 | 0.404 |

Supplementary Table 4. Model estimates and change relative to the control group mean for the sex sub-group analyses.

| Sub-group Analysis: Male, 8 trial arms | | | | | | | | | | | |
| --- | --- | --- | --- | --- | --- | --- | --- | --- | --- | --- | --- |
| Trial Group | Estimate | | Mean | | 95% CI (from exponentiated estimate) | | | | P-value | | Q-value |
| Control | NA | | 1418 | | NA | | NA | | NA | | NA |
| LARGE AND ADJACENT TO PRICE | 0.010 | | 1433 | | 1349 | | 1523 | | 0.740 | | 0.740 |
| LARGE AND ADJACENT TO PRODUCT | -0.018 | | 1394 | | 1314 | | 1477 | | 0.551 | | 0.630 |
| SMALL AND ADJACENT TO PRICE | -0.052 | | 1347 | | 1271 | | 1427 | | 0.078 | | 0.125 |
| SMALL AND ADJACENT TO PRODUCT | -0.032 | | 1373 | | 1292 | | 1460 | | 0.298 | | 0.397 |
| LP WITH SWITCH OFF FILTER | -0.068 | | 1325 | | 1247 | | 1407 | | 0.026 | | 0.071 |
| LP WITH SWITCH ON FILTER | -0.058 | | 1339 | | 1261 | | 1422 | | 0.060 | | 0.120 |
| LP WITH SUMMARY CALORIE LABEL | -0.069 | | 1324 | | 1247 | | 1405 | | 0.023 | | 0.071 |
| Male Sub-group Analysis: control versus all labelling trial arms combined | | | | | | | | | | | |
| Trial Group | Estimate | | Mean | | 95% CI (from exponentiated estimate) | | | | P-value | | Q-value |
| Control |  | | 1418 | | NA | | NA | | NA | | NA |
| ALL LABELLING TREATMENTS | -0.041 | | 1362 | | 1302 | | 1424 | | 0.073 | | 0.073 |
| Sub-group Analysis: Female, 8 trial arms | | | | | | | | | | | |
| Trial Group | | Estimate | | Mean | | 95% CI (from exponentiated estimate) | | | | P-value | Q-value |
| Control | | NA | | 1401 | | NA | | NA | | NA | NA |
| LARGE AND ADJACENT TO PRICE | | -0.052 | | 1330 | | 1257 | | 1407 | | 0.070 | 0.080 |
| LARGE AND ADJACENT TO PRODUCT | | -0.068 | | 1309 | | 1242 | | 1380 | | 0.011 | 0.018 |
| SMALL AND ADJACENT TO PRICE | | -0.028 | | 1362 | | 1288 | | 1440 | | 0.321 | 0.321 |
| SMALL AND ADJACENT TO PRODUCT | | -0.070 | | 1306 | | 1238 | | 1378 | | 0.010 | 0.018 |
| LP WITH SWITCH OFF FILTER | | -0.103 | | 1264 | | 1197 | | 1334 | | <0.001 | 0.001 |
| LP WITH SWITCH ON FILTER | | -0.099 | | 1268 | | 1202 | | 1338 | | <0.001 | 0.001 |
| LP WITH SUMMARY CALORIE LABEL | | -0.063 | | 1316 | | 1245 | | 1390 | | 0.025 | 0.033 |
| Female Sub-group Analysis: control versus all labelling trial arms combined | | | | | | | | | | | |
| Trial Group | | Estimate | | Mean | | 95% CI (from exponentiated estimate) | | | | P-value | Q-value |
| Control | |  | | 1401 | | NA | | NA | | NA | NA |
| ALL LABELLING TREATMENTS | | -0.069 | | 1308 | | 1256 | | 1362 | | 0.001 | 0.001 |

Supplementary Table 5. Model estimates and change relative to the control group mean for the eating disorder sub-group analyses.

| Sub-group Analysis: No eating disorder, control versus all labelling trial arms combined | | | | | | | | | | | |
| --- | --- | --- | --- | --- | --- | --- | --- | --- | --- | --- | --- |
| Trial Group | Estimate | Mean | | | 95% CI (from exponentiated estimate) | | | | P-value | | Q-value |
| Control |  | 1422 | | | NA | | | NA | NA | | NA |
| ALL LABELLING TREATMENTS | -0.033 | 1375 | | | 1299 | | | 1456 | 0.255 | | 0.255 |
| Sub-group Analysis: Non-binge eating disorder, control versus all labelling trial arms combined | | | | | | | | | | | |
| Trial Group | Estimate | | Mean | 95% CI (from exponentiated estimate) | | | | | P-value | | Q-value |
| Control |  | | 1393 | NA | | NA | | | NA | | NA |
| ALL LABELLING TREATMENTS | -0.031 | | 1351 | 1284 | | 1422 | | | 0.237 | | 0.237 |
| Sub-group Analysis: Binge eating disorder control versus all labelling trial arms combined | | | | | | | | | | | |
| Trial Group | Estimate | Mean | | | 95% CI (from exponentiated estimate) | | | | | P-value | Q-value |
| Control |  | 1430 | | | NA | | NA | | | NA | NA |
| ALL LABELLING TREATMENTS | -0.079 | 1322 | | | 1256 | | 1391 | | | 0.003 | 0.003 |

Supplementary Table 6. Model estimates and change relative to the control group mean for BMI sub-group analyses.

| Sub-group Analysis: Low BMI, 8 trial arms | | | | | | | | | | |
| --- | --- | --- | --- | --- | --- | --- | --- | --- | --- | --- |
| Trial Group | | Estimate | | Mean | | 95% CI (from exponentiated estimate) | | | P-value | Q-value |
| Control | | NA | | 1395 | | NA | | NA | NA | NA |
| LARGE AND ADJACENT TO PRICE | | -0.056 | | 1320 | | 1236 | | 1409 | 0.096 | 0.096 |
| LARGE AND ADJACENT TO PRODUCT | | -0.073 | | 1297 | | 1222 | | 1378 | 0.018 | 0.026 |
| SMALL AND ADJACENT TO PRICE | | -0.062 | | 1311 | | 1233 | | 1394 | 0.048 | 0.055 |
| SMALL AND ADJACENT TO PRODUCT | | -0.073 | | 1297 | | 1221 | | 1379 | 0.019 | 0.026 |
| LP WITH SWITCH OFF FILTER | | -0.104 | | 1257 | | 1181 | | 1338 | 0.001 | 0.003 |
| LP WITH SWITCH ON FILTER | | -0.111 | | 1248 | | 1173 | | 1328 | 0.000 | 0.002 |
| LP WITH SUMMARY CALORIE LABEL | | -0.098 | | 1265 | | 1188 | | 1347 | 0.002 | 0.005 |
| Low BMI Sub-group Analysis: control versus all labelling trial arms combined | | | | | | | | | | |
| Trial Group | | Estimate | | Mean | | 95% CI (from exponentiated estimate) | | | P-value | Q-value |
| Control | |  | | 1395 | | NA | | NA | NA | NA |
| ALL LABELLING TREATMENTS | | -0.082 | | 1285 | | 1226 | | 1345 | 0.0005 | 0.0005 |
| Sub-group Analysis: High BMI, 8 trial arms | | | | | | | | | | |
| Trial Group | Estimate | | Mean | | 95% CI (from exponentiated estimate) | | | | P-value | Q-value |
| Control | NA | | 1419 | | NA | | NA | | NA | NA |
| LARGE AND ADJACENT TO PRICE | 0.000 | | 1420 | | 1348 | | 1495 | | 0.997 | 0.997 |
| LARGE AND ADJACENT TO PRODUCT | -0.020 | | 1391 | | 1322 | | 1464 | | 0.442 | 0.589 |
| SMALL AND ADJACENT TO PRICE | -0.016 | | 1398 | | 1326 | | 1473 | | 0.563 | 0.643 |
| SMALL AND ADJACENT TO PRODUCT | -0.036 | | 1369 | | 1299 | | 1444 | | 0.184 | 0.369 |
| LP WITH SWITCH OFF FILTER | -0.060 | | 1337 | | 1269 | | 1409 | | 0.026 | 0.089 |
| LP WITH SWITCH ON FILTER | -0.056 | | 1342 | | 1275 | | 1413 | | 0.033 | 0.089 |
| LP WITH SUMMARY CALORIE LABEL | -0.029 | | 1379 | | 1310 | | 1452 | | 0.271 | 0.434 |
| High BMI Sub-group Analysis: control versus all labelling trial arms combined | | | | | | | | | | |
| Trial Group | Estimate | | Mean | | 95% CI (from exponentiated estimate) | | | | P-value | Q-value |
| Control |  | | 1419 | | NA | | NA | | NA | NA |
| ALL LABELLING TREATMENTS | -0.030 | | 1377 | | 1325 | | 1432 | | 0.126 | 0.126 |

Supplementary Table 7. Model estimates and change relative to the control group mean for frequency of delivery platform usage sub-group analyses, where low frequency platform users are defined as those that use the platform less than once a week and high frequency are those that use it more than once a week.

| Sub-group Analysis: Low Frequency Platform Users, 8 trial arms | | | | | | | |
| --- | --- | --- | --- | --- | --- | --- | --- |
| Trial Group | Estimate | Mean | 95% CI (from exponentiated estimate) | | | P-value | Q-value |
| Control |  | 1442 | NA | | NA | NA | NA |
| LARGE AND ADJACENT TO PRICE | -0.044 | 1380 | 1308 | | 1456 | 0.107 | 0.107 |
| LARGE AND ADJACENT TO PRODUCT | -0.071 | 1344 | 1278 | | 1414 | 0.006 | 0.010 |
| SMALL AND ADJACENT TO PRICE | -0.074 | 1339 | 1270 | | 1413 | 0.006 | 0.010 |
| SMALL AND ADJACENT TO PRODUCT | -0.071 | 1343 | 1272 | | 1418 | 0.010 | 0.012 |
| LP WITH SWITCH OFF FILTER | -0.092 | 1316 | 1248 | | 1387 | 0.001 | 0.002 |
| LP WITH SWITCH ON FILTER | -0.094 | 1313 | 1246 | | 1385 | 0.001 | 0.002 |
| LP WITH SUMMARY CALORIE LABEL | -0.070 | 1345 | 1275 | | 1419 | 0.011 | 0.012 |
| Low Freq. Platform Users Analysis: control versus all labelling trial arms combined | | | | | | | |
| Trial Group | Estimate | Mean | 95% CI (from exponentiated estimate) | | | P-value | Q-value |
| Control |  | 1442 | NA | | NA |  |  |
| ALL LABELLING TREATMENTS | -0.074 | 1340 | 1287 | | 1394 | 0.0003 | 0.0003 |
| Sub-group Analysis: High Frequency Platform Users, 8 trial arms | | | | | | | |
| Trial Group | Estimate | Mean | 95% CI (from exponentiated estimate) | | | P-value | Q-value |
| Control |  | 1375 | NA | NA | | NA | NA |
| LARGE AND ADJACENT TO PRICE | -0.001 | 1374 | 1291 | 1463 | | 0.987 | 0.987 |
| LARGE AND ADJACENT TO PRODUCT | -0.015 | 1355 | 1277 | 1437 | | 0.623 | 0.831 |
| SMALL AND ADJACENT TO PRICE | -0.001 | 1374 | 1294 | 1458 | | 0.974 | 0.987 |
| SMALL AND ADJACENT TO PRODUCT | -0.033 | 1330 | 1254 | 1411 | | 0.269 | 0.431 |
| LP WITH SWITCH OFF FILTER | -0.068 | 1285 | 1209 | 1366 | | 0.030 | 0.080 |
| LP WITH SWITCH ON FILTER | -0.068 | 1284 | 1211 | 1362 | | 0.024 | 0.080 |
| LP WITH SUMMARY CALORIE LABEL | -0.054 | 1303 | 1227 | 1383 | | 0.077 | 0.154 |
| High Freq. Platform Users Analysis: control versus all labelling trial arms combined | | | | | | | |
| Trial Group | Estimate | Mean | 95% CI (from exponentiated estimate) | | | P-value | Q-value |
| Control |  | 1375 | NA | NA | | NA | NA |
| ALL LABELLING TREATMENTS | -0.034 | 1329 | 1271 | 1390 | | 0.135 | 0.135 |

Supplementary Table 8. Model estimates and change relative to the control group mean for Task 2 exploratory analysis.

| Task 2: Contained Shop | | | | | | |
| --- | --- | --- | --- | --- | --- | --- |
| Trial Group | Estimate | Mean | 95% CI (from exponentiated estimate) | | P-value | Q-value |
| Control | NA | 862 |  |  | <0.00001 | <0.00001 |
| LARGE AND ADJACENT TO PRICE | -0.203 | 703 | 666 | 743 | <0.00001 | <0.00001 |
| LARGE AND ADJACENT TO PRODUCT | -0.230 | 685 | 650 | 721 | <0.00001 | <0.00001 |
| SMALL AND ADJACENT TO PRICE | -0.230 | 685 | 651 | 721 | <0.00001 | <0.00001 |
| SMALL AND ADJACENT TO PRODUCT | -0.190 | 713 | 676 | 751 | <0.00001 | <0.00001 |
| LP WITH SWITCH OFF FILTER | -0.205 | 703 | 667 | 740 | <0.00001 | <0.00001 |
| LP WITH SWITCH ON FILTER | -0.152 | 740 | 706 | 777 | <0.00001 | <0.00001 |
| LP WITH SUMMARY CALORIE LABEL | -0.200 | 706 | 669 | 744 | <0.00001 | <0.00001 |
| Task 2: control versus all labelling trial arms combined | | | | | | |
| Trial Group | Estimate | Mean | 95% CI (from exponentiated estimate) | | P-value | Q-value |
| Control |  | 862 | NA | NA | NA | NA |
| ALL LABELLING TREATMENTS | -0.201 | 705 | 682 | 729 | 0.000 | 0.000 |

Supplementary Table 9. Model estimates and change relative to the control group mean for exploratory analyses.

| Enjoyment Analysis: 8 trial arms | | | | | | | | |
| --- | --- | --- | --- | --- | --- | --- | --- | --- |
| Trial Group | Estimate | Mean | 95% CI | | | | | P-value |
| Control |  | 3.25 | NA | NA | | | | NA |
| LARGE AND ADJACENT TO PRICE | -0.047 | 3.20 | 3.20 | 3.30 | | | | 0.102 |
| LARGE AND ADJACENT TO PRODUCT | -0.045 | 3.21 | 3.21 | 3.30 | | | | 0.102 |
| SMALL AND ADJACENT TO PRICE | -0.010 | 3.24 | 3.28 | 3.30 | | | | 0.665 |
| SMALL AND ADJACENT TO PRODUCT | -0.019 | 3.23 | 3.26 | 3.30 | | | | 0.489 |
| LP WITH SWITCH OFF FILTER | -0.053 | 3.20 | 3.19 | 3.30 | | | | 0.102 |
| LP WITH SWITCH ON FILTER | -0.039 | 3.21 | 3.22 | 3.30 | | | | 0.129 |
| LP WITH SUMMARY CALORIE LABEL | -0.044 | 3.21 | 3.21 | 3.30 | | | | 0.102 |
| Enjoyment Analysis: control versus all labelling trial arms combined | | | | | | | | |
| Trial Group | Estimate | Mean | 95% CI (from exponentiated estimate) | | | | | P-value |
| Control |  | 3.25 | NA | NA | | | | NA |
| ALL LABELLING TREATMENTS | -0.036 | 3.22 | 3.18 | 3.25 | | | | 0.034 |
| Support Analysis: 8 trial arms | | | | | | | | |
| Trial Group | Estimate | Mean | 95% CI | | | | | P-value |
| Control |  | 2.74 | NA | | | NA | | NA |
| LARGE AND ADJACENT TO PRICE | 1.14 | 3.88 | 2.84 | | | 5.11 | | <0.001 |
| LARGE AND ADJACENT TO PRODUCT | 1.17 | 3.91 | 2.84 | | | 5.18 | | <0.001 |
| SMALL AND ADJACENT TO PRICE | 1.17 | 3.91 | 2.84 | | | 5.17 | | <0.001 |
| SMALL AND ADJACENT TO PRODUCT | 1.19 | 3.93 | 2.84 | | | 5.21 | | <0.001 |
| LP WITH SWITCH OFF FILTER | 1.16 | 3.90 | 2.84 | | | 5.16 | | <0.001 |
| LP WITH SWITCH ON FILTER | 1.28 | 4.02 | 2.84 | | | 5.39 | | <0.001 |
| LP WITH SUMMARY CALORIE LABEL | 1.20 | 3.94 | 2.84 | | | 5.23 | | <0.001 |
| Support Analysis: control versus all labelling trial arms combined | | | | | | | | |
| Trial Group | Estimate | Mean | 95% CI (from exponentiated estimate) | | | | | P-value |
| Control |  | 2.74 | NA | | | NA | | NA |
| ALL LABELLING TREATMENTS | 1.186 | 3.93 | 3.85 | | | 4.00 | | <0.001 |
| Calorie Awareness Analysis: 8 trial arms | | | | | | | | |
| Trial Group | Estimate | Mean | 95% CI | | | | | P-value |
| Control |  | 47.6 |  | | |  | | NA |
| LARGE AND ADJACENT TO PRICE | -23.0 | 24.5 | -54.5 | | | 103.6 | | 0.568 |
| LARGE AND ADJACENT TO PRODUCT | -91.6 | -44.0 | -118.8 | | | 30.7 | | 0.016 |
| SMALL AND ADJACENT TO PRICE | -56.5 | -8.9 | -86.7 | | | 68.9 | | 0.155 |
| SMALL AND ADJACENT TO PRODUCT | -30.5 | 17.0 | -62.0 | | | 96.0 | | 0.449 |
| LP WITH SWITCH OFF FILTER | -3.0 | 44.6 | -30.4 | | | 119.6 | | 0.938 |
| LP WITH SWITCH ON FILTER | -56.0 | -8.4 | -86.7 | | | 69.8 | | 0.161 |
| LP WITH SUMMARY CALORIE LABEL | -69.4 | -21.9 | -98.8 | | | 55.1 | | 0.077 |
| Calorie Awareness Analysis: control versus all labelling trial arms combined | | | | | | | | |
| Trial Group | Estimate | Mean | 95% CI (from exponentiated estimate) | | | | | P-value |
| Control |  | 47.554 | NA | | | NA | | NA |
| ALL LABELLING TREATMENTS | -48.1 | -0.551 | -59.59 | | | 58.49 | | 0.110 |
| Average Calories per Mains Selected Analysis: 8 trial arms | | | | | | | | |
| Trial Group | Estimate | Mean | 95% CI | | | | | P-value |
| Control |  | 924 | NA | | NA | | | NA |
| LARGE AND ADJACENT TO PRICE | 23.58 | 948 | 921 | | 974 | | | 0.082 |
| LARGE AND ADJACENT TO PRODUCT | 2.91 | 927 | 901 | | 953 | | | 0.825 |
| SMALL AND ADJACENT TO PRICE | -8.18 | 916 | 891 | | 942 | | | 0.529 |
| SMALL AND ADJACENT TO PRODUCT | -16.44 | 908 | 882 | | 934 | | | 0.220 |
| LP WITH SWITCH OFF FILTER | -6.49 | 918 | 891 | | 945 | | | 0.636 |
| LP WITH SWITCH ON FILTER | -29.22 | 895 | 869 | | 921 | | | 0.029 |
| LP WITH SUMMARY CALORIE LABEL | -4.08 | 920 | 894 | | 946 | | | 0.761 |
| Average Main Calories Analysis: control versus all labelling trial arms combined | | | | | | | | |
| Trial Group | Estimate | Mean | 95% CI (from exponentiated estimate) | | | | | P-value |
| Control |  | 924 | NA | | NA | | | NA |
| ALL LABELLING TREATMENTS | -5.82 | 918 | 899 | | 938 | | | 0.561 |
| Average Calories for a Restaurant Medium Main Analysis: 8 trial arms | | | | | | | | |
| Trial Group | Estimate | Mean | 95% CI | | | | | P-value |
| Control |  | 518 | NA | | | NA | | NA |
| LARGE AND ADJACENT TO PRICE | 12.16 | 530 | 507 | | | 553 | | 0.302 |
| LARGE AND ADJACENT TO PRODUCT | 10.46 | 528 | 506 | | | 551 | | 0.359 |
| SMALL AND ADJACENT TO PRICE | 11.19 | 529 | 507 | | | 551 | | 0.324 |
| SMALL AND ADJACENT TO PRODUCT | -8.64 | 509 | 488 | | | 531 | | 0.435 |
| LP WITH SWITCH OFF FILTER | 12.04 | 530 | 507 | | | 553 | | 0.308 |
| LP WITH SWITCH ON FILTER | -2.56 | 515 | 493 | | | 538 | | 0.821 |
| LP WITH SUMMARY CALORIE LABEL | 6.80 | 525 | 502 | | | 548 | | 0.559 |
| Average Calories for a Restaurant Medium Main Analysis: control versus all labelling trial arms combined | | | | | | | | |
| Trial Group | Estimate | Mean | 95% CI (from exponentiated estimate) | | | | | P-value |
| Control |  | 518 | NA | | | NA | | NA |
| ALL LABELLING TREATMENTS | 5.80 | 524 | 507 | | | 540 | | 0.492 |
| Average Portion Size Analysis: 8 trial arms | | | | | | | | |
| Trial Group | Estimate | Mean | 95% CI (from exponentiated estimate) | | | | | P-value |
| Control | 2.296 | 1.83 | NA | | | | NA | NA |
| LARGE AND ADJACENT TO PRICE | -0.004 | 1.82 | 1.77 | | | | 1.87 | 0.867 |
| LARGE AND ADJACENT TO PRODUCT | 0.032 | 1.86 | 1.81 | | | | 1.91 | 0.200 |
| SMALL AND ADJACENT TO PRICE | 0.016 | 1.84 | 1.79 | | | | 1.89 | 0.515 |
| SMALL AND ADJACENT TO PRODUCT | 0.015 | 1.84 | 1.79 | | | | 1.89 | 0.568 |
| LP WITH SWITCH OFF FILTER | -0.016 | 1.81 | 1.76 | | | | 1.86 | 0.535 |
| LP WITH SWITCH ON FILTER | 0.013 | 1.84 | 1.79 | | | | 1.89 | 0.618 |
| LP WITH SUMMARY CALORIE LABEL | 0.002 | 1.83 | 1.78 | | | | 1.88 | 0.925 |
| Average Portion Size Analysis: control versus all labelling trial arms combined | | | | | | | | |
| Trial Group | Estimate | Mean | 95% CI (from exponentiated estimate) | | | | | P-value |
| Control |  | 1.83 | NA | | | | NA | NA |
| ALL LABELLING TREATMENTS | 0.009 | 1.83 | 1.80 | | | | 1.87 | 0.638 |
| Number of Items Analysis: 8 trial arms | | | | | | | | |
| Trial Group | Estimate | Mean | 95% CI (from exponentiated estimate) | | | | | P-value |
| Control | 0.997 | 3.02 | NA | | NA | | | NA |
| LARGE AND ADJACENT TO PRICE | -0.057 | 2.85 | 2.75 | | 2.96 | | | 0.002 |
| LARGE AND ADJACENT TO PRODUCT | -0.051 | 2.87 | 2.77 | | 2.98 | | | 0.006 |
| SMALL AND ADJACENT TO PRICE | -0.049 | 2.88 | 2.77 | | 2.98 | | | 0.008 |
| SMALL AND ADJACENT TO PRODUCT | -0.029 | 2.93 | 2.82 | | 3.05 | | | 0.142 |
| LP WITH SWITCH OFF FILTER | -0.088 | 2.76 | 2.66 | | 2.87 | | | <0.001 |
| LP WITH SWITCH ON FILTER | -0.071 | 2.81 | 2.71 | | 2.92 | | | <0.001 |
| LP WITH SUMMARY CALORIE LABEL | -0.069 | 2.82 | 2.72 | | 2.93 | | | <0.001 |
| Number of Items Analysis: control versus all labelling trial arms combined | | | | | | | | |
| Trial Group | Estimate | Mean | 95% CI (from exponentiated estimate) | | | | | P-value |
| Control |  | 3.02 |  | |  | | | NA |
| ALL LABELLING TREATMENTS | -0.059 | 2.85 | 2.77 | | 2.93 | | | <0.001 |
| Items Removed Analysis: 8 trial arms | | | | | | | | |
| Trial Group | Estimate | Mean | 95% CI (from exponentiated estimate) | | | | | P-value |
| Control | -4.730 | 0.030 | NA | | NA | | | NA |
| LARGE AND ADJACENT TO PRICE | 0.007 | 0.030 | 0.018 | | 0.048 | | | 0.977 |
| LARGE AND ADJACENT TO PRODUCT | 0.019 | 0.030 | 0.019 | | 0.048 | | | 0.937 |
| SMALL AND ADJACENT TO PRICE | -0.237 | 0.024 | 0.014 | | 0.039 | | | 0.364 |
| SMALL AND ADJACENT TO PRODUCT | -0.192 | 0.025 | 0.015 | | 0.040 | | | 0.462 |
| LP WITH SWITCH OFF FILTER | 0.159 | 0.035 | 0.022 | | 0.055 | | | 0.516 |
| LP WITH SWITCH ON FILTER | -0.661 | 0.016 | 0.009 | | 0.027 | | | 0.025 |
| LP WITH SUMMARY CALORIE LABEL | -0.151 | 0.026 | 0.016 | | 0.042 | | | 0.560 |
| Items Removed Analysis: control versus all labelling trial arms combined | | | | | | | | |
| Trial Group | Estimate | Mean | 95% CI (from exponentiated estimate) | | | | | P-value |
| Control |  | 0.029711 | NA | | NA | | |  |
| ALL LABELLING TREATMENTS | -0.133 | 0.026116 | 0.018 | | 0.037 | | | 0.479 |

**Appendix**

Survey questions for participants

Demographic and screening questions:

1. [Age] How old are you?
   1. Numeric input; 18 to 99 eligible
2. [Gender] Are you…
   1. [Male / Female / Other]
3. [Ethnicity] What is your ethnic group?
   1. [White (Includes British, Northern Irish, Irish, Gypsy, Irish Traveller, Roma, or any other White background) / Mixed or Multiple ethnic groups (Includes White and Black Caribbean, White and Black African, White and Asian, or any other Mixed or Multiple background) / Asian or Asian British (Includes Indian, Pakistani, Bangladeshi, Chinese, or any other Asian background) / Black, Black British, Caribbean or African (Includes Black British, Caribbean, African, or any other Black background / Other ethnic group (Includes Arab or any other ethnic group)]
4. [Region] In which region do you live?
   1. [East Midlands (England) / East Of England / London / North East (England) / North West (England) / Northern Ireland / Scotland / South East (England) / South West (England) / Wales / West Midlands (England) / Yorkshire And The Humber]
5. [Income] What is your current annual household income before taxes?
   1. [Less than £5,000 / £5,000 to £9,999 / £10,000 to £14,999 / £15,000 to £17,499 / £17,500 to £19,999 / £20,000 to £22,499 / £22,500 to £24,999 / £25,000 to £27,499 / £27,500 to £29,999 / £30,000 to £32,499 / £32,500 to £34,999 / £35,000 to £37,499 / £37,500 to £39,999 / £40,000 to £42,499 / £42,500 to £44,999 / £45,000 to £47,499 / £47,500 to £49,999 / £50,000 to £54,999 / £55,000 to £59,999 / £60,000 to £64,999 / £65,000 to £69,999 / £70,000 to £74,999 / £75,000 to £99,999 / £100,000 and above / Prefer not to answer]
6. [Education] What is the highest education level that you have achieved?
   1. [Less than high school / High school completed / University degree / None of the above]
7. [BMI] Please specify:
   1. [height] your height
   2. [weight] your weight

(Two numerical input boxes separately for height and weight, labelled as e.g. cm / inches; kg / lbs. Coded such that only one of the answer boxes can be filled by the participant for each)

1. [SES] What is the current profession of the chief income earner in your household?*
   *That's the person with the highest income, whether that be from employment, pensions, state benefits, investment or other sources.
   1. [Higher managerial/ professional/ administrative (e.g. Established doctor, Solicitor, Board Director in a large organisation (200+ employees), top level civil servant/public service employee) / Intermediate managerial/ professional/ administrative (e.g. Newly qualified (under 3 years) doctor, Solicitor, Board director small organisation, middle manager in large organisation, principal officer in civil service/local government) / Supervisory or clerical/ junior managerial/ professional/ administrative (e.g. Office worker, Student Doctor, Foreman with 25+ employees, salesperson, etc) / Student / Skilled manual worker (e.g. Skilled Bricklayer, Carpenter, Plumber, Painter, Bus/ Ambulance Driver, HGV driver, AA patrolman, pub/bar worker, etc) / Semi or unskilled manual work (e.g. Manual workers, all apprentices to be skilled trades, Caretaker, Park keeper, non-HGV driver, shop assistant) / Casual worker – not in permanent employment / Housewife/ Homemaker / Retired and living on a state pension / Unemployed or not working due to long-term sickness / Full-time career of other household member]
2. [OrderFrequency] On average, how often do you order from food delivery platforms, like Deliveroo, JustEat, UberEats etc.?
   1. [Every day / A few times a week / Once a week / Once a month / Less than once a month / **Never - ineligible: exclude**]

Attention check questions:

1. People are very busy these days and some do not properly read survey questions. To show that you've read this much, answer both "Extremely interested" and "Very interested."
   1. [Not interested at all / Slightly interested / Moderately interested / Very interested / Extremely interested]
2. [asked if participant fails the first attention check] You didn’t select the correct answers to our last question. Your attention to the survey questions is very important for our research, so we’d like to give you another chance to respond. To show that you are paying attention, answer both "Extremely interested" and "Very interested."
   1. [Not interested at all / Slightly interested / Moderately interested / Very interested / Extremely interested]

Questions after Task 1:

1. [Enjoyment] How pleasant or unpleasant was your shopping experience?
   1. 1=very unpleasant / 2=unpleasant/ 3=pleasant/ 4=Very pleasant
2. If [Enjoyment] == 1 or 2 - Why was your shopping experience unpleasant? [Free text]
3. If [Enjoyment] == 3 or 4 - Why was your shopping experience pleasant? [Free text]
4. How many calories do you think you ordered in the simulated shopping task?
   1. Numerical box of integer numbers. Participants can choose between ‘kcal’ or ‘kJ’.
5. [CaloriePreference] Do you prefer energy information to be presented using:
   1. Kilocalorie (kcal)
   2. Kilojoules (kJ)
   3. Both

Questions after Task 2:

1. When thinking about delivery apps, how much would you oppose or support following practice on food delivery apps? [followed by the description of the randomised intervention/control] (Please note, for simplicity the description will be in the form of an annotated screenshot of the intervention or the control)
   1. 1=Strongly oppose / 2=Oppose / 3= Neither oppose nor support/ 4=Support / 5=Strongly support
2. Why do you oppose/support this? [free text]
3. If it was entirely up to you, what would be your ideal weight?
   1. [Two numerical input boxes separately for height and weight, labelled as e.g. cm / inches; kg / lbs. Coded such that only one of the answer boxes can be filled by the participant for each]
4. Please pick a response for each of the following statements:
   1. I eat diet foods
   2. I feel extremely guilty after eating.
   3. I think about burning up calories when I exercise.
   4. I feel uncomfortable after eating sweets.
   5. I find myself preoccupied with food.
   6. I am terrified about being overweight.
   7. I am preoccupied with a desire to be thinner.
   8. I am preoccupied with the thought of having fat on my body
   9. Have gone on eating binges where I feel that I may not be able to stop.

1 = I agree & I agree somewhat / 0 = I disagree & I disagree somewhat / 99 = Prefer not to say
